# Corrigenda in the Proemium

**Published:** 1820-03

**Authors:** 


### CORRIGENDA IN THE PROEMIUM.

Page xvii. line 8, after *Life* add an *asterisk* (*). P. xx. *dele* the first line of the note (§) all but the last three words. P. xxi. l. 20, for *sanguineous* read *venous;* same page, *dele* the note (§); and, same page, l. 30, for *his* read *Legallois*; same page, note (‡), for *this* read *these.* P. xxv. note (*), for 40*th* and 42*d*, read 41*st* and 43*d*. P. xxviii. l. 40, before *dogmas* add *of the.* P. xxxiv. five lines from the bottom, for *affect* read *effect.* P. xl. l. 26, for *opinion* read *opinions;* same page, note (*) make one word of *bes chouwd* (thus, *beschouwd*); same note, for *al* read *als.* P. lvi. which begins with *Dr. Bricheteau*, l. 2, dele *and.*—(N.B. The paging from this part to lxxxvi. is incorrect, but the matter of the work runs correctly). The next page, marked lxv. last line but one of note (*), for *his* read *this.* P. lxxi. in the paragraph on the epidemic varioloid disease, for *reports* read *results.* The next page, six lines from the bottom, for *results* read *result.* Two pages onwards, marked lxvi. l. 31, after *opinion* add *has been.* Next page, lxvii. note (*), for *euhonic* read *euphonic.* P. lxviii. note, for *chevrot* read *chevre.* P. lxxiii. (terminating *semiology*) line 10, for *his* read *this.* P. lxxix. line 13, for *the* read *a*; same page, note (**), for *nan* read *van*, and for *aes* read *als.* P. lxxxvii. line 7, for *found* read *formed.* P. lxxxix. last line, for *grows* read *grow.* P. xc. five lines from the bottom, for *pistil* read *pistils.* P. xci. line 33, dele *into*; same page, last line but one, for *poatoe* read *potatoe.* P. xcv. line 3, for *has* read *have.* Last p. note (*) dele the *s* in *theoriques*; for *d'etat* read *l'etat.*

This list of errors, principally typographical, will appear very long. The reader should be informed, that the *Proëmium* was written in a fortnight, and passed the press in a very few days; and the author writes a character that is not legible without difficulty by persons unaccustomed to it, even when most *leisurely penned.*

